# Medical Society of the State of Wisconsin

**Published:** 1853-08

**Authors:** 


### EDITORIAL.

*Medical Society of the State of Wisconsin.*

MESSRS. EDITORS:

With this note I mail you a copy of the Law of Wisconsin, touching medical societies, etc., that you may better understand the minutes herewith furnished. You will notice, if you take the trouble to look, that our State Medical Society is composed of permanent members, and of delegates from County and District Societies; and that only two persons can become permanent members in any one year. The delegates from the local societies are chosen annually, one, for every five members.

This law was enacted in the year 1841, and has been in operation since.

Now, after an existence of twelve years, look at the State Medical Society, and what is its condition? Indeed, most wretched! The most that appears on inspection of the books, is a bare record of the names of members, a large majority of whom never attend a meeting of the society, unless held in their own town, and not always then. This is a sad picture to present, but it is too true to be controverted.

Of the several local societies, not one is in a prosperous condition—at least we may so conclude from the fact, that, at the last annual meeting of the State Society, only three permanent members were present, and *not one delegate!* Well, the couclusion is natural, either that the Law is worthless, or that the medical profession in this state is miserably lifeless—in either case it behooves the profession to be up and doing; for, if the fault be in the law, let us have a new enactment, or act entirely independent of legislative aid, and if the fault be in the profession, the sooner it is corrected the better.

Therefore it is, gentlemen, that I would, through your columns, most respectfully urge upon the medical profession throughout Wisconsin, the importance of taking hold of this matter in earnest, for there is much work to be done, and only a few "regulars" to do it. Look you! is it in other parts of the state as in this, namely, *more than three quacks, to every regularly educated physician?* If so, is there nothing to be done? This, however, is only one item on the list of "things to be done."

Then, let every "regular" feel it incumbent on him, to come up to the work and be "on hand" at Madison, on the fourth day of January A. D. 1854. If all do this, it is yet hoped that there is sufficient ability and energy in the profession of this state, to sustain, henceforth, an efficient medical organization.

But, gentlemen, I must not detain you longer—I have done.

Yours cordially

GEO. D. WILBER.

Mineral Point, Wis.,

July 14, 2853,

The Medical Society of the State of Wisconsin met at Janesville on Tuesday, the 14th of last June, to hold its annual session; but there being no quorum present, the society adjourned to meet at Madison on the fourth Wednesday of Jan. A.D. 1854.

Attest

A. L. CASTLEMAN,
*Pres. pro tem.*

GEO. D. WILBER,
*Cor. Sect'y.*

